# *CG31320* (*Heatr2*) - ciliopathy candidate gene, functional analysis in fly and mouse models

**DOI:** 10.1186/2046-2530-1-S1-P92

**Published:** 2012-11-16

**Authors:** G Mali, P Mill, PI zur Lage, EA Hall, AP Jarman, IJ Jackson

**Affiliations:** 1 MRC Human Genetics Unit, MRC IGMM, University of Edinburgh, UK; 2 University of Edinburgh, UK

## 

The structural and functional roles of many of the 800-1000 proteins that make up the microtubule core and specialized membranes of cilia and flagella are poorly understood. Following from our recent expression study to identify putative ciliary candidates in *Drosophila* sensory neurons, we focused on a subset that were targets of the transcription factor Fd3f, which regulates functional specialization of mechanosensory cilia. Bioinformatic enrichment for known ciliary domains as well as orthologous protein-protein interaction network modelling provided a list of putative ciliary genes for further functional characterization. One such candidate, *CG31320*, has been initially characterized in *Drosophila*. Little is known about this gene, except the encoded protein contains HEAT repeats – belonging to an armadillo-like fold family associated with intracellular transport. In situ analysis confirms that *CG31320* mRNA is highly expressed in the ciliated chordotonal neurons. RNAi-mediated knock-down resulted in abnormal chordotonal ciliary morphology and locomotory defects, consistent with impaired mechanosensory cilium function. Currently, we are studying whether the ortholog Heatr2 is also required for mammalian cilia. Protein localization studies suggest that Heatr2 plays a role in trafficking to primary cilia. RNAi knock-down and protein interaction studies using mammalian cells are underway to functionally dissect Heatr2 roles; results will be presented. We are generating a *Heatr2* conditional mouse mutant to investigate its function in different types of cilia and sperm flagella. We present a multisystem experimental pipeline for functional characterization of novel genes expressed in cilia as well as putative ciliopathy candidates.

